# Deposition Mechanism and Characterization of Plasma-Enhanced Atomic Layer-Deposited SnO*_x_* Films at Different Substrate Temperatures

**DOI:** 10.3390/nano12162859

**Published:** 2022-08-19

**Authors:** Pao-Hsun Huang, Zhi-Xuan Zhang, Chia-Hsun Hsu, Wan-Yu Wu, Sin-Liang Ou, Chien-Jung Huang, Dong-Sing Wuu, Shui-Yang Lien, Wen-Zhang Zhu

**Affiliations:** 1 School of Ocean Information Engineering, Jimei University, Jimei District, Xiamen 361021, China; 2 School of Opto-Electronic and Communication Engineering, Xiamen University of Technology, Xiamen 361024, China; 3 Department of Materials Science and Engineering, National United University, Miaoli 36063, Taiwan; 4 Department of Biomedical Engineering, Da-Yeh University, Changhua 51591, Taiwan; 5 Department of Applied Physics, National University of Kaohsiung, Kaohsiung University Road, Kaohsiung 81148, Taiwan; 6 Department of Applied Materials and Optoelectronic Engineering, National Chi Nan University, Nantou 54561, Taiwan; 7 Department of Materials Science and Engineering, Da-Yeh University, Changhua 51591, Taiwan

**Keywords:** tin oxide (SnO*_x_*), plasma-enhanced atomic layer deposition (PEALD), substrate temperature

## Abstract

The promising functional tin oxide (SnO*_x_*) has attracted tremendous attention due to its transparent and conductive properties. The stoichiometric composition of SnO*_x_* can be described as common n-type SnO_2_ and p-type Sn_3_O_4_. In this study, the functional SnO*_x_* films were prepared successfully by plasma-enhanced atomic layer deposition (PEALD) at different substrate temperatures from 100 to 400 °C. The experimental results involving optical, structural, chemical, and electrical properties and morphologies are discussed. The SnO_2_ and oxygen-deficient Sn_3_O_4_ phases coexisting in PEALD SnO*_x_* films were found. The PEALD SnO*_x_* films are composed of intrinsic oxygen vacancies with O-Sn^4+^ bonds and then transformed into a crystalline SnO_2_ phase with increased substrate temperature, revealing a direct 3.5–4.0 eV band gap and 1.9–2.1 refractive index. Lower (<150 °C) and higher (>300 °C) substrate temperatures can cause precursor condensation and desorption, respectively, resulting in reduced film qualities. The proper composition ratio of O to Sn in PEALD SnO*_x_* films near an estimated 1.74 suggests the highest mobility of 12.89 cm^2^ V^−1^ s^−1^ at 300 °C.

## 1. Introduction

Transparent conductive oxide (TCO) materials have been widely used and intensively researched in a wide range of industries during the last half-century [1,2,3,4]. Because of its increasing use in many instruments, this large area of constantly expanding research has focused on the preparations and properties of TCO films such as tin oxide (SnO_2_), indium tin oxide (ITO), zinc oxide (ZnO), aluminum-doped ZnO (AZO), and titanium oxide (TiO_2_) [5,6,7,8,9]. Non-stoichiometric SnO_2_ (SnO*_x_*), in particular, has recently gained substantial interest as a potential functional oxide semiconductor for use in a wide range of optoelectronics due to its specific features in its stoichiometry [10]. For instance, the SnO*_x_* films are prepared with nanocomposite porous silicon for application and used in gas microsensors [11]. Due to their superior chemical and mechanical stability over other known oxide films, SnO*_x_* films are also employed as electron selective film candidates for solar cells and light-emitting diodes based on perovskite, quantum dots, and organic materials [12,13,14]. Furthermore, various experiments have been conducted to examine tin oxides with different oxygen stoichiometry, such as Sn_2_O_3_ [15], Sn_3_O_4_ [16], and Sn_5_O_6_ [17]. Because of its oxygen-deficient property, p-type Sn_3_O_4_ has gained a significant amount of interest. However, the impact of this Sn_3_O_4_ phase on the optical, electrical, physical, and chemical characteristics of the film, which usually coexists with the SnO_2_ phase, is sometimes underestimated.

In the literature, various deposition processes such as chemical vapor deposition (CVD), low-pressure chemical vapor deposition, plasma-enhanced chemical vapor deposition (PECVD), physical vapor deposition (PVD), and so on have been employed to prepare multifunctional SnO*_x_* films [18,19,20,21]. Currently, atomic layer deposition (ALD), as an appealing deposition process with low deposition temperature, atomic-scale thickness controllability, and remarkable conformity, permits the considerable scaling-down and 3D structuring of devices as compared to CVD and PVD [22,23]. In ALD, two self-limiting surface reactions are used, in which two reactant gases are pulsed into the chamber in two different dosages, resulting in the formation of individual mono-layers per reaction cycle. Furthermore, as a better approach, plasma-enhanced ALD (PEALD) employs plasma-generated oxidizing agents to effectively augment the reactivity between plasma species and precursors, allowing for lower deposition temperatures without affecting film quality [24,25,26,27]. Film properties are affected by different deposition modes driven by lower or higher substrate temperatures [28,29,30], perhaps due to precursor condensation/adsorption within an incomplete reaction or decomposition/desorption. Thus, it is important to focus on the impact of various substrate temperatures on the PEALD SnO*_x_* films and validate which deposition mode will occur with the various substrate temperatures in order to acquire the optimal stoichiometry of oxygen and tin.

PEALD SnO*_x_* films deposited at substrate temperatures ranging from 100 to 400 °C are investigated in this study. The metal precursor is tetrakis(dimethylamino)tin (TDMA-Sn), which reacts with oxygen and argon plasma reactants. The optical, electrical, physical, and chemical characteristics are analyzed and discussed to determine the optimal stoichiometric ratio of O to Sn.

## 2. Materials and Methods

### 2.1. Materials and PEALD Process

The SnO*_x_* films were deposited on silicon wafers (4 inches with 450 μm and a resistivity of 50 Ω-cm) by the PEALD system (R-200, Picosun, Finland) with six source channels, where the TDMA-Sn (purity: 99.9999%, Aimou Yuan, Nanjing, China) was used as the Sn metal precursor. Each experimental variable was used for preparing five samples at different substrate temperatures, and silicon wafers were cleaned by a standard procedure, including deionized water (DI-water) for 10 s, hydrofluoric acid for 1 min, and DI water for 10 s. Before being transferred to the vacuum chamber, the silicon wafer was blow-dried with nitrogen (N_2_, 99.99%). We operated the Ar and O_2_ (both of them with an ultra-high purity of 99.999%) plasma in a quartz cavity by the inductive coupling of RF power. The SnO*_x_* deposition was performed with a total of 300 ALD cycles. Table 1 shows the preparation parameters of the PEALD SnO*_x_* films, and the substrate temperature was varied from 100 to 400 °C.

### 2.2. Characteristic Measurements

The ellipsometer (M-2000, J. A. Woollan Co., Lincoln, NE, USA) was used to determine the thickness, refractive index (*n*), and deposition rate (nm/cycle). The estimated thickness value had an error of less than ±2% to show satisfying reproducibility. The model of “air, air/SnO*_x_*, SnO*_x_*, SnO*_x_*/silicon” was used to complete the fitting ellipsometric data for the PEALD SnO*_x_* films by the Drude-Lorentz model. For the optical properties of films, all samples were measured by ultraviolet-visible spectroscopy (MFS-630, Hong-Ming Technology, New Taipei City, Taiwan) in the wavelength range from 350 to 850 nm. For the structural properties of films, the grazing incidence X-ray diffraction (XRD, Rigaku TTRAXIII, Ibaraki, Japan) with a selected 0.5° incident angle and a wavelength of 0.15418 nm was used at 50 kV and 300 mA to obtain the orientation in diffraction patterns within a 2θ range of 20° to 70°. Field emission scanning electron microscopy (FESEM, JSM-7800F, JEOL, Tokyo, Japan) at 9.6 × 10^−5^ Pa and atomic force microscopy (AFM, XE7, Park, Korea) at ambient conditions were used to obtain the top-view surface morphologies. Further microstructure characteristics were shown in the cross-sectional transmission electron microscopy (TEM) images. For the chemical properties of the films, the X-ray photoelectron spectroscopy (XPS, ESCALAB, 250Xi, Thermo Fisher, Waltham, MA, USA) spectra were performed and calibrated by C 1s (284.5 eV). Before XPS measurement, the surface contamination was removed by sputtering. For the electrical properties of films, the resistivity, carrier concentration, and mobility were conducted by Hall-effect measurements (HMS-5000, Side Semiconductor Technology, Ecopia, Anyang, Korea) at room temperature. Both XRD and XPS results were further analyzed by peak-differentiated and imitating methods to demonstrate the phase and bonding characteristics of the films, respectively.

## 3. Results and Discussion

### 3.1. Deposition Mechanism

**Figure 1:**
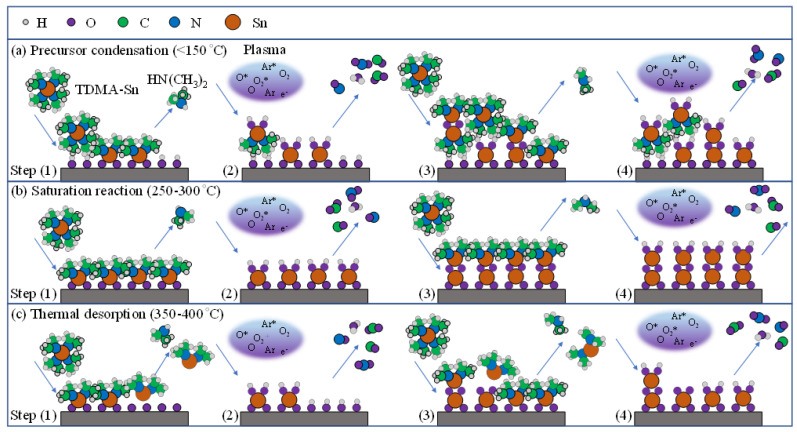
Deposition mechanism of PEALD SnO*_x_* films at different substrate temperatures dividing into three growth modes: (**a**) precursor condensation (<150 °C), (**b**) saturation reaction (250–300 °C), and (**c**) thermal desorption (350–400 °C).

The schematic deposition mechanism of the PEALD SnO*_x_* films is shown in Figure 1. Three growth modes concerning the first (steps 1 and 3) and second self-limiting surface reactions (steps 2 and 4) are described as (a) precursor condensation (<150 °C), (b) saturation reaction (250–300 °C), and (c) thermal desorption (350–400 °C), where the reaction can be represented via the following equations [28,29]:

*S^*-(OH)_3_ + 2 Sn(N(CH_3_)_2_)_4_ → *S^*-OH-Sn(N(CH_3_)_2_)_4_ + *S^*-O_2_Sn(N(CH_3_)_2_)_2_ + 2 NC_2_H_7_↑ (1a)

*S^*-(OH)_2_ + Sn(N(CH_3_)_2_)_4_ → *S^*-O_2_Sn(N(CH_3_)_2_)_2_ + 2 NC_2_H_7_↑ (1b)

*S^*-(OH)_2_ + Sn(N(CH_3_)_2_)_4_ → *S^*-O_2_Sn(N(CH_3_)_2_)_2_ + NC_2_H_7_↑ + Sn(NC_2_H_7_)_2_↑ (1c)

*S^*-O_2_Sn(N(CH_3_)_2_)_2_ + *Plasma* (O*/Ar*/e^−^) → *S^*-SnO_2_-H + (CO*_X_* + NO*_X_* + H_2_O)↑ (2)

*S^*-SnO_2_-2H + Sn(N(CH_3_)_2_)_4_ → *S^*-SnO_2_-Sn(N(CH_3_)_2_)_2_ + NC_2_H_7_↑ (3)

*S^*-SnO_2_-Sn(N(CH_3_)_2_)_2_ + *Plasma* (O*/Ar*/e^−^) → *S^*-SnO_2_-SnO_2_-2H + (CO*_X_* + NO*_X_* + H_2_O)↑ (4)

where the *S^* and ↑ symbols represent the substrate surface and by-product with volatile gaseous phase, respectively. In Equation (1), the TDMA-Sn molecules will react with the hydroxyl (OH) groups on the substrate surface. Equation (1a) reveals that the low substrate temperature (<150 °C) causes the condensation of the TDMA-Sn precursor mainly due to the physisorption, where it is quite mobile and oscillating on the surface. This result is similar to some other studies [28]. With the increasing substrate temperatures (200–400 °C), the physisorption becomes a minor factor and the film growth gradually turns into chemisorption as a significant factor. As shown in Equations (1b) and (1c), the thermal activation induces the irreversible break of chemical bonding and the electron transfer between the deposited surface and adsorbed molecules [31,32]. Notably, when the substrate temperature is in the range of 250–300 °C, a self-limiting PEALD process emerges as Equation (1b) due to enough heat energy, leading to the saturation reaction of precursors and oxygen radicals. However, these adsorbed precursor molecules will further desorb, as in Equation (1c), when the surface possesses excess heat energy at higher substrate temperatures of 350–400 °C. In the second self-limiting surface reaction, the plasma reaction is shown as the following formula to generate oxygen (O_2_) radicals: Ar + O_2_ + e^−^→2O* + Ar* + e^−^, where the asterisk mark describes the excited state. The Sn-O bonds and initial hydroxyl ligands are formed, and then the released by-products (CO*_X_*, NO*_X_*, and H_2_O gas), as described in Equation (2), are purged. So far, one PEALD cycle has finished, and we continuously used more than one cycle to complete the film growth by repeating Equations (3) and (4).

**Figure 2:**
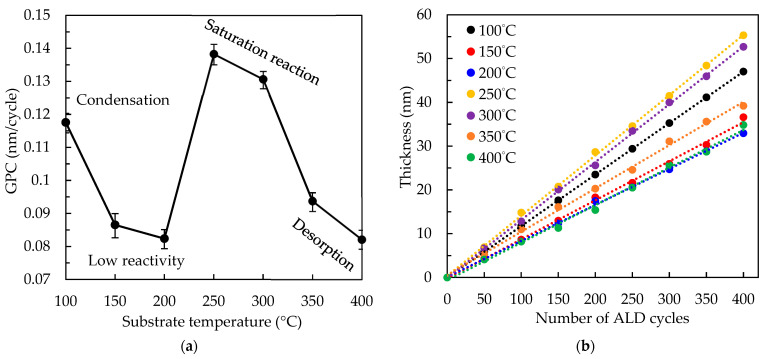
(**a**) The substrate temperature-dependent growth per cycle (GPC) of PEALD SnO*_x_* films and (**b**) its trend line of corresponding thickness at each GPC.

Figure 2a shows the substrate temperature-dependent growth per cycle (GPC) of PEALD SnO*_x_* films on the Si wafer from 100 to 400 °C. The trend line of corresponding thickness at each GPC is plotted in Figure 2b. We calculate the GPC value by dividing the film thickness by the number of cycles. Three reaction regions are obviously demonstrated with respect to the substrate temperature. The GPC of 0.117 nm/cycle at 100 °C is mainly induced by the precursor physisorption and condensation [28,33]; however, at 150–200 °C, the GPC decreases to 0.117–0.087 nm/cycle, inferring that the surface reaction changes from physisorption to chemisorption-dominated. These low GPC values are likely due to the low chemical reaction rates at low temperatures [34,35]. The high GPC values of 0.138 nm/cycle at 250 °C and 0.131 nm/cycle at 300 °C are ascribed to the saturation of chemical-adsorbed precursors. However, the GPC rapidly drops to 0.094 nm/cycle at 350 °C and 0.082 nm/cycle at 400 °C due to the severe thermal desorption between the precursors and surface [34]. In other words, the self-limiting process as a unique feature of PEALD is verified by observing the saturation reaction of the GPC value as a function of the substrate temperature. Compared to some studies contrary to our results [28], these observations indicate that the higher substrate temperature causes the low GPC owing to the precursor’s desorption [34]. In the ALD process, the saturation reaction should lead to a relatively high GPC value and simultaneously a small change in GPC, which were observed in the range of 250 to 300 °C in this study. This temperature range is reasonable as compared to the literature [36].

### 3.2. Chemical and Electronic State of the Sn and O

**Figure 3:**
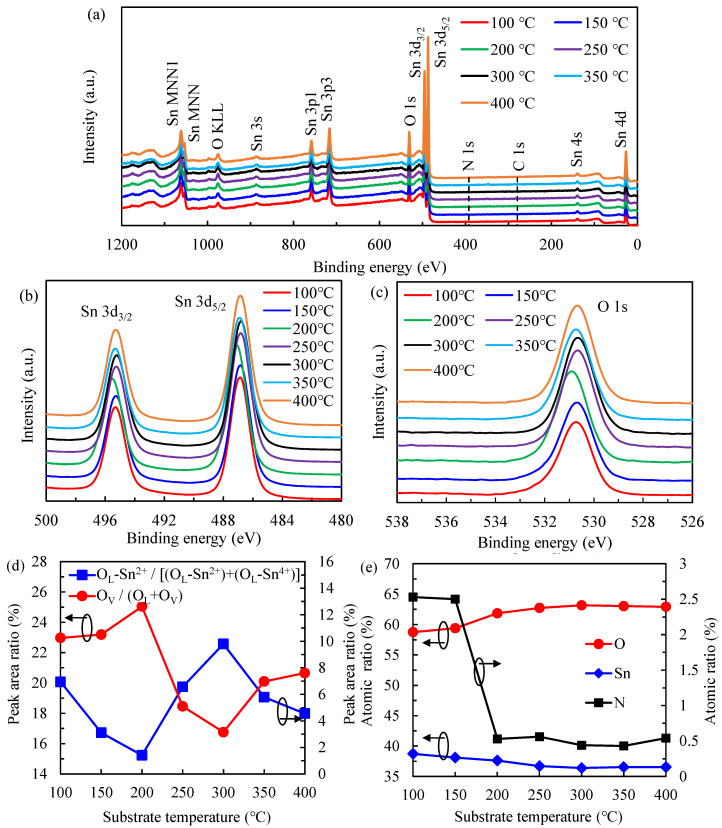
(**a**) XPS spectrums for the PEALD SnO*_x_* films deposited at substrate temperatures from 100 and 400 °C. The spectra of (**b**) Sn 3d and (**c**) O 1s core level with (**d**) the peak area ratio of O_L_–Sn^2+^/[(O_L_–Sn^2+^) + (O_L_–Sn^4+^)] and O_V_/(O_L_ + O_V_), and (**e**) the atomic ratio of O, Sn, and N elements.

Figure 3a shows the XPS full-side spectra for the films deposited at different temperatures. All the peaks are labeled and hydrogen is not detectable in XPS, while nitrogen and carbon may be contained in the films, but only in low amounts. The nitrogen content of around 2.5 at.% at 100 and 150 °C results from the unreacted ligands of TDMA-Sn, possibly due to the low reactivity at low substrate temperatures. At higher substrate temperatures (>200 °C), the nitrogen content is as low as around 0.5 at.%. In particular, Sn 3d_3/2_ and 3d_5/2_ peak at ~495.6 and~487.0 eV [37], respectively, and the O 1s peak at ~530.7 eV is commonly used for further analysis. In Figure 3b, showing the high-resolution Sn 3d peaks, the peak position is slightly different among the samples with different substrate temperatures. This is related to the Sn^4+^ and Sn^2+^ components, e.g., at respectively 487.5 eV and at 486.4 eV for the Sn5/2 peaks [38]. The Sn^4+^ and Sn^2+^ components indicate the coexistence of the SnO_2_ and the metastable Sn oxide (such as Sn_3_O_4_). This is also supported by the O 1s spectra illustrated in Figure 3c. The spectra are deconvoluted into three peaks at 530.0 eV, associated with the lattice oxygen bonded to Sn^2+^ (O_L_–Sn^2+^); 531 ± 0.1 eV, to the lattice oxygen bonded to Sn^4+^ (O_L_–Sn^4+^); and 532 ± 0.1 eV to oxygen-deficient regions in oxides [24,39,40]. The ratio of each oxygen component to the total is calculated and shown in Figure 3d. At low temperatures (100–200 °C) the O_L_–Sn^2+^ area ratio decreases from 20.07% to the lowest of 15.23%, and the oxygen vacancy (O_V_) defects proportion increases from 10.25% to the highest of 12.62%, primarily due to the precursor chemisorption dominating at 200 °C.

The maximum 22.57% O_L_–Sn^2+^ area ratio and the minimum 3.15% O_V_ proportion at 300 °C are observed. This suppression of O_V_ defects is mainly due to the best decomposition of the precursor at 300°C. Besides, the O_V_ defects proportion increases to 7.61% at 400 °C due to the out-diffusion of the oxygen atoms from SnO_2_ films. It is deduced that at higher substrate temperatures, the SnO_2_ decomposes thermally and oxygen breaks bonds between itself and metal and diffuses towards the film surface. The oxygen then leaves the film as O_2_, and it is possible that a small amount of oxygen leaves the film as CO_2_. The atomic ratios of elemental compositions, including O, Sn, and nitrogen (N), as a function of substrate temperature, are shown in Figure 3e. Notably, the high N ratio of ~2.5% at 100 °C and 150 °C dramatically decreases to ~0.5% in the range of 200–400 °C, demonstrating that the precursors are decomposed above 200°C. To analyze the stoichiometric SnO*_x_* films, the O to Sn ratio values (R_O/Sn_) are further calculated at different substrate temperatures. The R_O/Sn_ of 1.517 at 100 °C increases to 1.559 and 1.645 at 150 °C and 200 °C, respectively. Then, the improved R_O/Sn_ is obtained as 1.709 at 250 °C and 1.736 at 300 °C. The excessively high temperatures (350 °C and 400 °C) show a slightly decreased R_O/Sn_ of 1.725 and 1.723, respectively. These results are similar to a few studies [37].

### 3.3. Structural Properties of the SnO_x_ film

**Figure 4:**
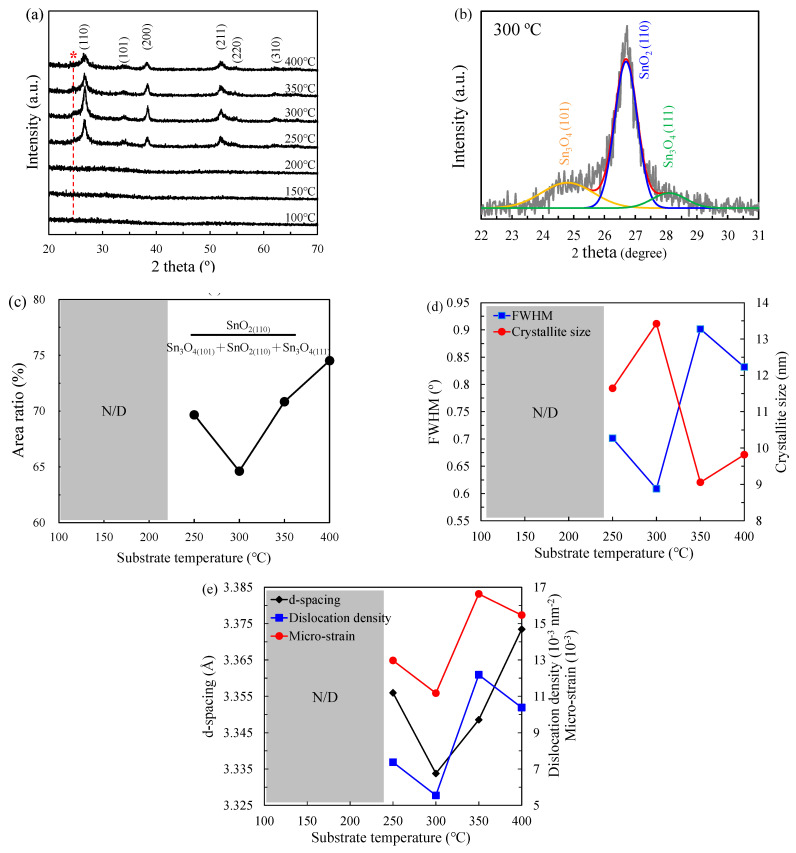
(**a**) XRD patterns of PEALD SnO*_x_* films deposited at substrate temperatures where the red star mark with red dash line presents another (101) orientation of Sn_3_O_4_. (**b**) The variation for the FWHM of the preferential (110) orientation and the average crystallite size, showing (**c**) the dependence of the average dspacing of (110) planes, the dislocation density, and the micro-strain value. (**d**) The deconvolution results of the (110) orientation deposited at 300 °C in the 2 theta of 22–31°. (**e**) The variation of the area ration of (110)_SnO2_ to [(110)_SnO2_ + (101)_Sn3O4_ + (111)_Sn3O4_].

Figure 4a illustrates the XRD patterns of PEALD SnO*_x_* films deposited at different substrate temperatures. Based on the JCPDS card (no. 41-1445), the strong peaks at 26.7°, 38.4°, and 52.1° are ascribed to (110), (200), and (211) orientations of the SnO_2_ tetragonal rutile structure, respectively [24,25,41]. The weak peaks at 34.2°, 53.1°, and 62.4° correspond to (101), (220), and (310) orientations, respectively [27]. The amorphous structure of films deposited at below 200 °C is clearly observed. The reason is the low reactivity of precursor and precursor condensation induced by the low substrate temperature. With the increasing substrate temperatures, a polycrystalline SnO_2_ is observed. A (110) preferred orientation is detected with the highest intensity variability when the substrate temperature is in the range of 250–400 °C. The intensity of (110) orientation increases at medium temperature (250–300 °C) due to the self-limiting growth and then decreases at higher substrate temperature (350–400 °C), owing to the decomposition and desorption of the precursor. The intensity variation of diffraction peaks indicates the consistent variation of full width at half maximum (FWHM). Figure 4b shows the FWHM variation of the preferential (110) orientation and the average crystallite size (*D*) of films estimated by the Scherrer function as Equation (5) [42]:

*D* = κ*λ*/(*β*cos *θ*), (5)

where the κ = 0.9 is the Scherrer constant, *λ* is the wavelength of the X-ray sources, *β* is the FWHM value, and *θ* as Bragg angle is the peak position of the (110) orientation. The lowest FWHM value of 0.87° at 300 °C corresponds to the largest average crystallite size. Then, the FWHM value increases with increasing substrate temperature from 300 °C to 350 °C, indicating the decreased average crystallite size from 13.42 to 9.06 nm. The reason is attributed to the fact that excessively high substrate temperature above 300 °C causes the non-ideal deposition induced by severe precursor desorption and decomposition. It is observed that the diffraction peaks slightly shift with the increasing substrate temperature, suggesting a lattice expansion or contraction. For example, the peak position shifts from 26.56° at 250 °C to 26.74° at 300 °C. Similarly, the peak position then shifts toward a lower angle to 26.42° at 400 °C. The interplanar distance (*d*−spacing) is calculated as shown in Figure 4c by the Bragg formula [43]:

2*d*sin *θ* = *n_d_λ*, (6)

where *n_d_* is the order of diffraction, and *d* is the dspacing. With increasing substrate temperatures from 250 °C to 400 °C, the dspacings of SnO*_x_* films are around 3.356, 3.334, 3.349, and 3.373 Å, respectively. The standard dspacing value of pure SnO_2_ is 3.347 Å. The decreased dspacing when increasing the substrate temperature from 250 to 300 °C is attributed to the decrease in oxygen vacancy defects as observed from the XPS results, causing the lattice contraction of SnO*_x_* films [44]. In the study reported by Santara et al. [45], the oxygen interstitials (O_i_^2+^) and metal interstitials may attract each other and cause lattice contraction. Thus, another possible reason for the lattice contraction observed in this study can be due to the electrostatic attraction between O_i_^2+^ and tin interstitials (Sn_i_^4+^). In contrast, the increased d−spacings at 350–400 °C are due to the generated oxygen vacancy defects. The O−Sn bonds in the vicinity of oxygen-deficient regions are relaxed, leading to the lattice expansion of SnO*_x_* films. Besides, the nearest-neighbor Sn atoms move outward from the vacancy to strengthen their neighboring bonds of the remaining oxygen lattice. Although the nearest-neighbor oxygen atoms may move inward to fill the site of oxygen vacancy defects, the net outward movement of Sn atoms is higher than the net inward movement of oxygen atoms, resulting in the lattice expansion. Other microstructural parameters, such as micro-strain (*ɛ*) and dislocation density (*δ*), are estimated as:

***ε*** = *β/*4tan *θ*, (7)

*δ* = 1*/D*^2^, (8)

Accordingly, the film at the 300 °C substrate temperature obtains the lowest value of *δ* and *ɛ*. The small *δ* obtained at 300 °C is the number of defects measured in the crystals [43] and the released *ε* at 250–300 °C is mainly due to the lattice contraction. The enhanced *ε* at 300–350 °C can be described by the increased vacancy formation energy from external strain [46].

However, beyond the substrate temperature of 300 °C, we observe that the (110) SnO_2_ peaks are not symmetrical, possibly implying the existence of other phases. For example, a diffraction peak near 25° is observed as a star, marked in Figure 4a, resulting from the oxygen-deficient SnO*_x_* [27]. To identify whether there are other hidden peaks, the (110) peaks are deconvoluted in Figure 4d, where two shoulder peaks at 24.8° and 28.1° as (101) and (111) triclinic Sn_3_O_4_ phases are identified (JCPDS#16-0737) [47,48]. This means that the SnO*_x_* films have SnO_2_ as the major phase and Sn_3_O_4_ as the minor phase. Moreover, the triclinic Sn_3_O_4_ as an intermediate oxide during the phase transformation of SnO_2_ is known as the oxygen-deficient SnO*_x_* phase [49]. The (110)_SnO2_/[(110)_SnO2_ + (101)_Sn3O4_ + (111)_Sn3O4_] peak area ratio for the different substrate temperatures is further shown in Figure 4e. The proportion of (110) orientation firstly decreases to the lowest value of 64.62% at 300 °C and then increases again at increasing substrate temperatures. This result also supports that the 300 °C substrate temperature is a critical temperature where the deposition mode changes from saturation growth to precursor decomposition or desorption.

**Figure 5:**
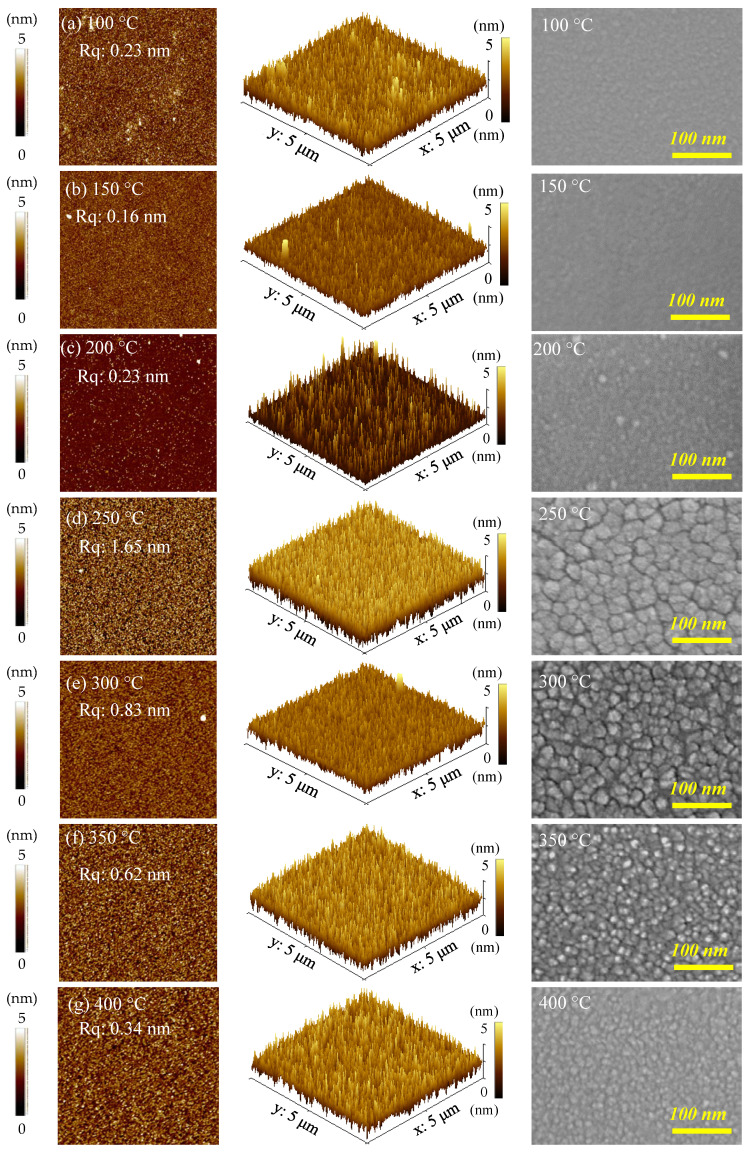
Topographic and stereoscopic surface morphologies of AFM with a scanning area of 5 × 5 µm^2^ and top-view images of FESEM for PEALD SnO*_x_* films deposited at various substrate temperatures from (**a**–**g**) 100 °C to 400 °C on a Si wafer.

AFM with a scanning area of 5 × 5 µm^2^ is used to analyze the topographic and stereoscopic surface morphologies of PEALD SnO*_x_*, films as shown in Figure 5. The films grown at 100–200 °C show a smooth microstructure with a root-mean-square (Rq) of 0.16–0.23 nm, consistent with the amorphous SnO*_x_* films at this temperature range. The film deposited at 250 °C obtains the highest Rq value of 1.65 nm. The Rq reduces to 0.34 nm when the substrate temperature increases to 400 °C. Compared to the Rq value of SnO*_x_* films deposited by spray pyrolysis (11.6 nm) [50] and sputtering (17.72 nm) [51], the PEALD SnO*_x_* films provide a smoother surface that is beneficial for many applications.

The top-view FESEM images of the films are observed on the right-hand side of Figure 5. Flat and featureless morphologies of SnO*_x_* films are observed without noticeable grain boundaries at the substrate temperature of 100–200 °C. This agrees with the amorphous structure of the films. At 250 °C, distinct clusters can be observed due to the large SnO*_x_* grains, and a clear grain structure is visible at 300 °C; however, these obvious grain boundaries gradually disappear at the higher substrate temperatures of 350 °C and 400 °C, attributed to the decreased grain size.

**Figure 6:**
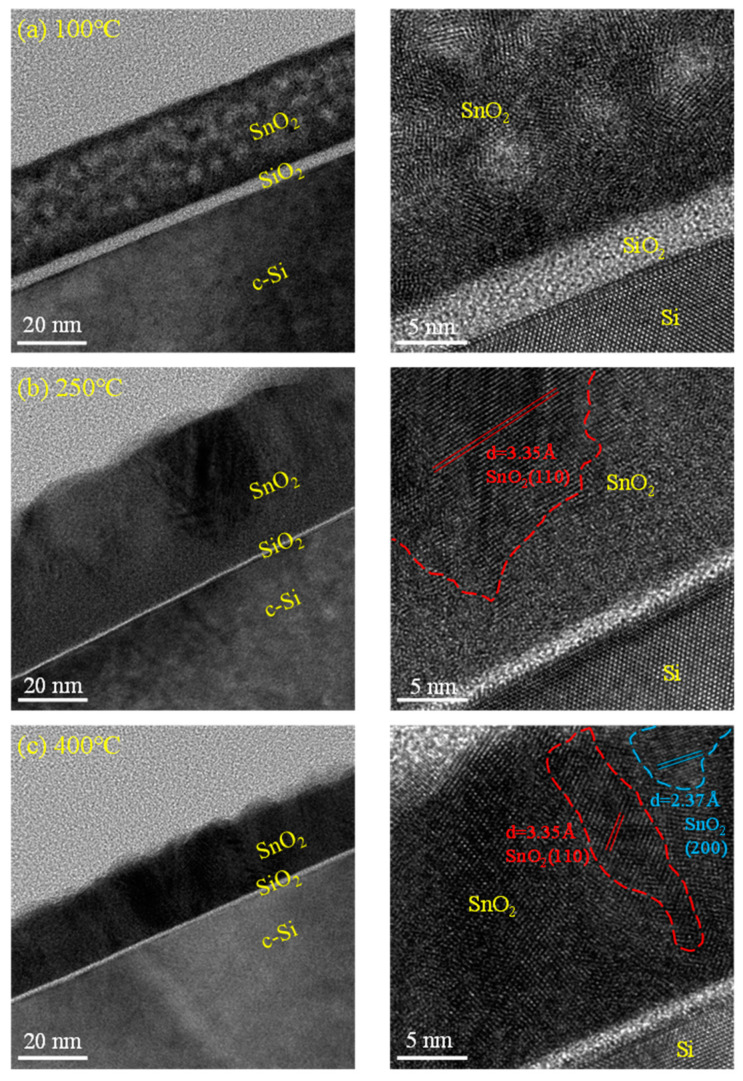
The TEM results of SnO*_x_* films deposited at different substrate temperatures of (**a**) 100 °C, (**b**) 250 °C, and (**c**) 400 °C, including cross-sectional and high-resolution images.

Figure 6 shows the cross-sectional TEM images of SnO*_x_* films. It is unexpected that crystallization is observed at 100 °C, shown in Figure 6a, as this is inconsistent with the XRD result. One reasonable explanation is that the amorphous structure recrystallizes by the ion beam of the TEM measurement.

In Figure 6b, the 41.49 nm-thick SnO*_x_* film deposited at 250 °C reveals well-defined lattice fringes with a dspacing of 3.35 Å corresponding to the (110) SnO_2_ tetragonal rutile structure. The film deposited at 400 °C shown in Figure 6c shows lattice fringes of 2.3 and 3.35 Å dspacings corresponding to SnO_2_ (200) and (110) planes. At the Si/SnO*_x_* interface, the silicon oxide layer is presented, and its thickness decreases from 3.9 (100 °C) to 1.5 nm (400 °C). The presence of the interfacial layer is similar to our previous research of ALD HfO_2_ or Al_2_O_3_ [52,53], and thus the reason is believed to be attributed to the reaction between oxygen plasma radicals and the Si wafer in the first few cycles.

### 3.4. Photoelectric Properties of the SnO_x_ film

**Figure 7:**
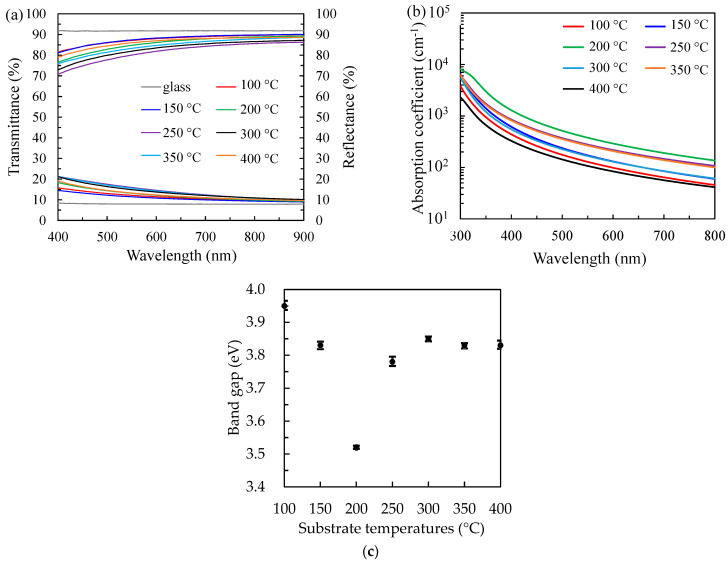
(**a**) The optical transmittance with reflectance and (**b**) absorption coefficient spectrums to extract (**c**) the band gap values for the PEALD SnO*_x_* films with increasing substrate temperatures from 100 to 400 °C.

Figure 7a shows the optical spectra of PEALD SnO*_x_* films deposited at different substrate temperatures. The variation of the transmittance spectrum is inverse to that of reflectance. All samples have a transmittance of approximately 80% to 90% and a reflectance of approximately 10% to 15% in the wavelength range of 400–900 nm. The decrease in transmittance at the short wavelength of around 400 nm for the films is attributed to the absorption caused by the band-to-band transition. In addition, the absorption coefficient (*α*) is determined by the Beer–Lambert law equation [31,33]:

α = 4π*k/λ*, (9)

where *λ* is the wavelength and *k* is the extinction coefficient determined from ellipsometer measurements. The absorption coefficients are further used for the optical band gap determination using Tauc’s plot method [54]:

(αh*ν*)^2^ = A·(h*ν* − Eg), (10)

where h*ν* is the photon energy and A is the proportionality constant [55]. As shown in Figure 7c, Eg with the V-shaped trend on the substrate temperature is observed. With the increasing substrate temperatures, the SnO*_x_* film obtains the narrowest Eg of 3.52 eV at 200 °C, possibly due to the introduction of a shallow donor energy level of oxygen vacancies (O_V_) under the conduction band [56,57]. Another reason is the smaller excited energy induced by the short-range ordered crystallite in the amorphous SnO*_x_* crystal, leading to the increase in the carrier concentration [58,59]. Furthermore, the enhancement of Eg to 3.78 eV is obtained at 250°C, mainly owing to the presence of polycrystalline, as evidenced in XRD results. The Eg increases slightly from 3.78 to 3.85 eV at 250–300 °C and then maintains 3.83 eV at 350 °C and 400 °C.

**Figure 8:**
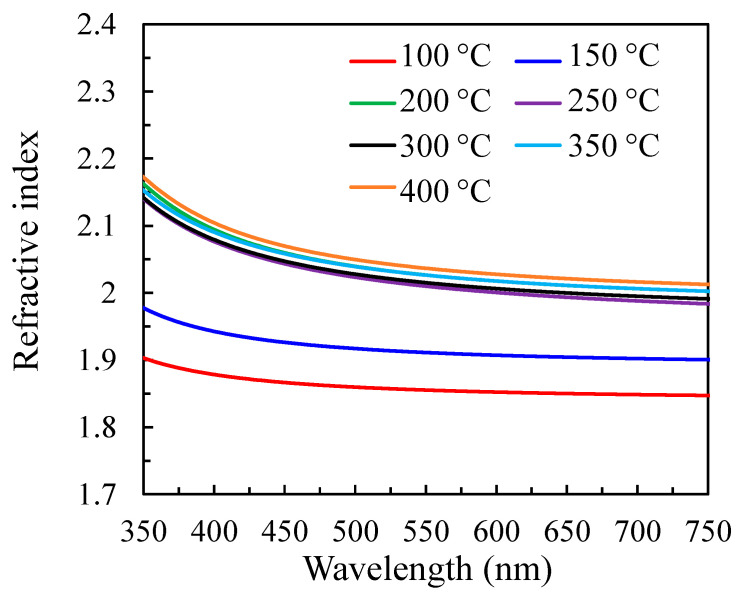
The wavelength-dependent refractive index of PEALD SnO*_x_* films with increasing substrate temperatures from 100 to 400 °C.

Figure 8 demonstrates the wavelength-dependent refractive index of the PEALD SnO*_x_* films with different substrate temperatures. The refractive index is low for the samples at 100–200 °C, then varies closely at 250–350 °C, and reaches the highest value at 400 °C. This variation of the refractive index can be a reflection of the change in the film density, since they are closely related [25]. Increasing the substrate temperature from 100 to 200 °C causes the increase in packing density in the amorphous structure and the change in the chemical composition of the films (especially nitrogen proportion), hence affecting the refractive index. Meanwhile, the variation of refractive index at high substrate temperatures (250–400 °C) also corresponds to the crystallinity variation in the polycrystalline structure.

**Figure 9:**
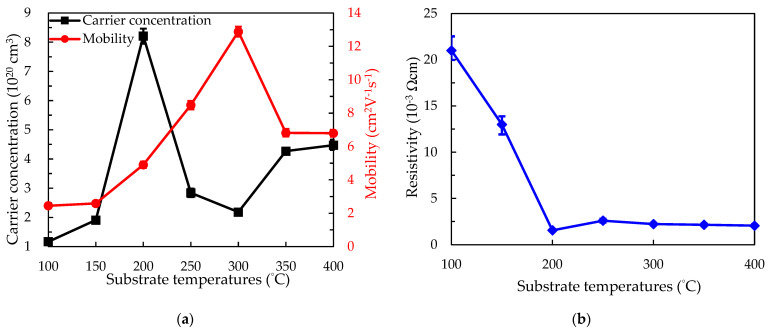
Hall-effect measurement for the (**a**) carrier concentration (*Ne*) accompanied by the mobility (*µ*) and (**b**) resistivity (*ρ*) of SnO*_x_* films deposited at various substrate temperatures at 100–400 °C. Five samples were measured in each series, and standard deviations are included.

As a transparent conductive material, the electrical properties are an important indicator for PEALD SnO*_x_* films. As a result, the carrier concentration (*Ne*), mobility (*μ*), and resistivity (*ρ*) of PEALD SnO*_x_* films were determined by Hall-effect measurements. In Figure 9a, the films deposited at 100°C obtained the lowest *Ne* of 1.17 × 10^20^ cm^−3^ and *μ* of 2.44 cm^2^/Vs. With the increasing substrate temperatures, the *Ne* slightly increases to 1.91 × 10^20^ cm^−3^ at 150 °C and sharply sweeps upward to the highest 8.22 × 10^20^ cm^−3^ at 200 °C. After that, we observed that the *Ne* descends to 2.84 × 10^20^ cm^−3^ at 250 °C and even 2.18 × 10^20^ cm^−3^ at 300 °C. The possible reason for this trend is attributed to the variation of crystallization and the proportion of oxygen vacancies, where the change presents similar consistency to Figure 3d. Upon increasing the substrate temperature to 350 °C, the increased proportion of oxygen vacancies becomes the main reason for the suddenly increased *Ne* to 4.27 × 10^20^ cm^−3^. However, we have noticed that, primarily, the μ gradually ascends with the increasing substrate temperature from 100 °C to 300 °C, maybe owing to an enhancement of the crystallinity of the films and thus electrical continuity in the lateral direction [28]. Higher substrate temperatures cause the decreased *μ* at 350 °C and 400 °C due to the variation of crystallinity and crystallite size in SEM results, resulting from the phase transition during the deposition. Figure 9b shows the high *ρ* values of SnO*_x_* films below 150°C determined by the *Ne* and the low *μ*. Low *ρ* values at 200–400 °C are shown in the range of 1.5 to 2.6 × 10^−3^ Ω·cm.

## 4. Conclusions

In this work, PEALD SnO*_x_* films were prepared at various substrate temperatures, and their optical, physical, and chemical properties were further studied. The deposition mechanisms associated with three temperature ranges are clearly demonstrated. The precursor condensation is observed at low substrate temperatures (100–200 °C), forming the amorphous structure with the highest carrier concentration of 8.22 × 10^20^ cm^−3^. The surface reaction at 200 °C changes from physisorption to chemisorption-dominated. Meanwhile, the precursors are largely decomposed to participate in the reaction due to the dramatic decrease in the N ratio. With the increasing substrate temperatures, the PEALD SnO*_x_* films prepared at 250–400 °C show the coexistence of SnO_2_ and Sn_3_O_4_ phases. The lowest (110) SnO_2_ ratio is obtained at 300 °C. However, the film prepared at the substrate temperature of 300 °C has the highest O_L_−Sn^2+^ and the lowest O_V_ ratios. The excessive 350 °C and 400 °C initiated severe precursor desorption, leading to a decrease in the GPC and mobility. The ratio of O to Sn at 300 °C is further estimated to be ~1.74 as a preferred parameter for depositing high-quality PEALD SnO*_x_* films.

## Figures and Tables

**Table 1:** Preparation parameters of PEALD SnO_2_ films.

Parameter	Value
Bubbler temperature (°C)	50
Substrate temperature (°C)	100–400
TDMA-Sn pulse time (s)	1.6
TDMA-Sn purge time (s)	6
O_2_ pulse time (s)	11
O_2_ purge time (s)	5
Ar flow rate (sccm)	80
O_2_ flow rate (sccm)	150
O_2_ plasma power (W)	2000
TDMA-Sn carry gas flow rate (sccm)	120
TDMA-Sn dilute gas flow rate (sccm)	400

## Data Availability

Not applicable.
